# Influence of Polyformaldehyde Monofilament Fiber on the Engineering Properties of Foamed Concrete

**DOI:** 10.3390/ma15248984

**Published:** 2022-12-15

**Authors:** Md Azree Othuman Mydin, Mohd Mustafa Al Bakri Abdullah, Mohd Nasrun Mohd Nawi, Zarina Yahya, Liyana Ahmad Sofri, Madalina Simona Baltatu, Andrei Victor Sandu, Petrica Vizureanu

**Affiliations:** 1 School of Housing, Building and Planning, Universiti Sains Malaysia, Gelugor 11800, Penang, Malaysia; 2 Faculty of Chemical Engineering & Technology, Universiti Malaysia Perlis, Arau 01000, Perlis, Malaysia; 3 Centre of Excellence Geopolymer and Green Technology (CEGeoGTech), Universiti Malaysia Perlis (UniMAP), Arau 01000, Perlis, Malaysia; 4 Disaster Management Institute (DMI), School of Technology Management and Logistics, Universiti Utara Malaysia, Sintok 06010, Kedah, Malaysia; 5 Department of Technologies and Equipments for Materials Processing, Faculty of Materials Science and Engineering, Gheorghe Asachi Technical University of Iaşi, Blvd. Mangeron, No. 51, 700050 Iasi, Romania; 6 Romanian Inventors Forum, Str. Sf. P. Movila 3, 700089 Iasi, Romania; 7 National Institute for Research and Development in Environmental Protection INCDPM, Splaiul Independentei 294, 060031 Bucharest, Romania; 8 Technical Sciences Academy of Romania, Dacia Blvd 26, 030167 Bucharest, Romania

**Keywords:** foamed concrete, polypropylene fibrillated fiber, compression, flexural, tensile, porosity, water absorption

## Abstract

Foamed concrete is considered a green building material, which is porous in nature. As a result, it poses benefits such as being light in self-weight, and also has excellent thermal insulation properties, environmental safeguards, good fire resistance performance, and low cost. Nevertheless, foamed concrete has several disadvantages such as low strength, a large amount of entrained air, poor toughness, and being a brittle material, all of which has restricted its usage in engineering and building construction. Hence, this study intends to assess the potential utilization of polypropylene fibrillated fiber (PFF) in foamed concrete to enhance its engineering properties. A total of 10 mixes of 600 and 1200 kg/m^3^ densities were produced by the insertion of four varying percentages of PFF (1%, 2%, 3%, and 4%). The properties assessed were splitting tensile, compressive and flexural strengths, workability, porosity, water absorption, and density. Furthermore, the correlations between the properties considered were also evaluated. The outcomes reveal that the foamed concrete mix with 4% PFF attained the highest porosity, with approximately 13.9% and 15.9% for 600 and 1200 kg/m^3^ densities in comparison to the control specimen. Besides, the mechanical properties (splitting tensile, compressive and flexural strengths) increased steadily with the increase in the PFF percentages up to the optimum level of 3%. Beyond 3%, the strengths reduced significantly due to poor PFF dispersal in the matrix, leading to a balling effect which causes a degraded impact of scattering the stress from the foamed concrete vicinity to another area of the PFF surface. This exploratory investigation will result in a greater comprehension of the possible applications of PFF in LFC. It is crucial to promote the sustainable development and implementation of LFC materials and infrastructures.

## 1. Introduction

The construction industry around the world has become the industry that utilizes the most energy, recording approximately fifty percent of the total amount of energy used, as urbanization has progressed, and more people have moved into cities. In the context of global climate change, the construction industry is mulling over an alternative for normal-strength concrete as a means of mitigating the extreme self-weight of the material as well as the substantial carbon discharge connected with the production of cement [1]. As a result, the implementation of environmentally friendly and energy-saving building materials as an alternative to conventional building materials has become the industry standard [2]. The porous nature of foamed concrete, which is a green building material, confers upon it several advantages, including its low cost, excellent thermal insulation properties, environmental protections, and excellent fire resistance performance [3]. Compared to normal-strength concrete, foamed concrete has a higher strength-to-weight ratio and a bulk density that ranges from 500 to 1850 kg/m^3^ [4]. This distinction results in a decrease in the total dead load of the structural components, as well as a decrease in the costs of manufacturing and labor during the transportation and construction processes [5].

Foamed concrete is broadly applied in the construction industry as a filling material, as a non-loadbearing wall constituent, and as insulation material for exterior panels [6]. To deliver this porous cellular form of foamed concrete, small air bubbles of differing sizes are first inserted into the newly blended materials [7]. This is typically accomplished through the use of a foaming method that is either chemical or mechanical [8]. In its most basic form, the material component that goes into the production of foamed concrete is identical to that of normal-strength concrete, which consists of Portland cement, aggregate, and water [9]. This is despite the fact that the fabrication of foamed concrete only requires the use of fine sand. In addition, lightweight filler or reinforcement such as rice husk ash or polypropylene fiber can be combined with the foamed concrete base mix to expand its range of characteristics [10]. 

In spite of this, it is commonly believed that foamed concrete has poor physical, mechanical, and durability properties, which is principally due to its high porosity and void connectivity, both of which allow harmful substances to enter foamed concrete media [11]. In addition, foamed concrete has some disadvantages, including low strength, a high volume of entrapped air, poor toughness, and brittle material, which have limited its use in engineering and building construction [12]. These restrictions have decreased the use of foamed concrete [13]. As a result, it was determined that foamed concrete should not be used for principal load-bearing structural elements. Less than five percent of global use statistics were attributable to the construction industry’s utilization of foamed concrete. This study investigated the use of PFF to improve the engineering qualities of foamed concrete in order to counteract its negative tendencies. Steel fiber-reinforced concrete is extensively utilized in a wide range of civil engineering projects [14] due to its low cost, high performance, and ease of production. 

Despite this, a few studies have demonstrated that the uneven inclusion of steel fiber in concrete can have a negative effect on the workability of the fresh mix, leading to porous concrete and a reduction in the strength parameter [15,16]. By incorporating fibrous components into the mix, the characteristics of the concrete can be improved. The tensile and shear capacities of concrete can be enhanced by adding polypropylene fiber, according to past studies [17,18]. It is feasible for polypropylene to raise the strength of a material without increasing its dry bulk density [19]. According to some evidence, the application of propylene fiber membrane marginally increases the engineering qualities of concrete, specifically its tensile strength [20]. Compared to thick fibers, a membrane constructed of propylene fiber plays a key function in reducing plastic shrinkage [21]. 

Polypropylene fibrillated fiber (PFF), one of the synthetic fibers, has the potential to be utilized as an additive in foamed concrete to improve its qualities. All the PFF membrane’s exceptional mechanical qualities, alkali resistance, and thermal properties contribute to its superior performance. In addition, the PFF membrane is acknowledged as a fiber that may be incorporated into a cement-based composite material which has a high resistance to cracking. The incorporation of PFF into concrete permits the formation of a three-dimensional dispersion network, which effectively suppresses the development of microcracks in cement-based materials. 

On the basis of the preceding review, it can be concluded that the influence of PFF inclusion in foamed concrete on the enhancement of engineering qualities has not been explored and discussed in detail. Consequently, the fundamental objective of this study is to determine the engineering characteristics of foamed concrete, reinforced with a PFF membrane. By altering the weight percentages of PFF in the production process, densities of 600 kg/m^3^ and 1200 kg/m^3^ were achieved. 

## 2. Materials and Methods

### 2.1. Mix Constituents

**Figure 1:**
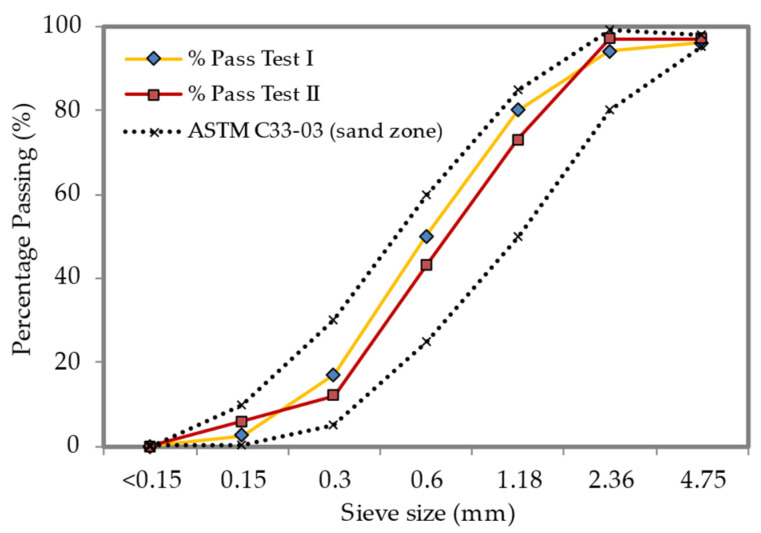
Sand grading curve of fine river sand with ASTM-C33 upper and lower limits.

**Figure 2:**
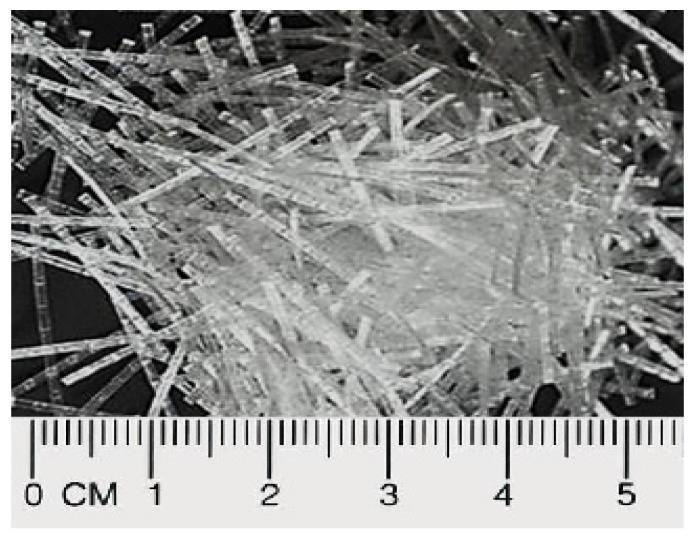
Polypropylene fibrillated fiber (PFF).

The primary materials used in this study were fine river sand that had been sieved through a 600-micron sieve with a specific gravity of 2.59 g/cm^3^ and particle sizes ranging from 0.15 to 2.36 mm, as well as Portland cement that complied with the specifications of the British Standards Institution (1996). The entire required fine river sand quantity underwent a sieve examination to fit the coarse aggregate standard specification, as per what was stated in ASTM-C33. Figure 1 shows the result of the sand grading curve. Besides, using a protein-based surfactant, stable foam with a density of 70 kg/m^3^ was produced. It was attenuated in the mortar slurry at a ratio of 1:34 and then aerated using a TM-1 foam generator. Then, four different weight fractions of polypropylene fibrillated fiber (Figure 2) in a range from 1% to 4% were added to the foamed concrete mixtures. Table 1 displays the physical and mechanical characteristics of polypropylene fibrillated fiber (PFF).

### 2.2. Mix Proportions

In this investigation, densities of 600 and 1200 kg/m^3^ were cast. The cement-to-sand ratio was held at 1:1.5, and the water-to-cement proportion was maintained at 0.45 for all mixtures. Five distinct PFF weight fractions of 1%, 2%, 3% and 4% were selected for addition to foamed concrete mixtures. Table 2 displays the proportions of the created foamed concrete mixture.

## 3. Results

### 3.1. Flow Table Test

**Figure 3:**
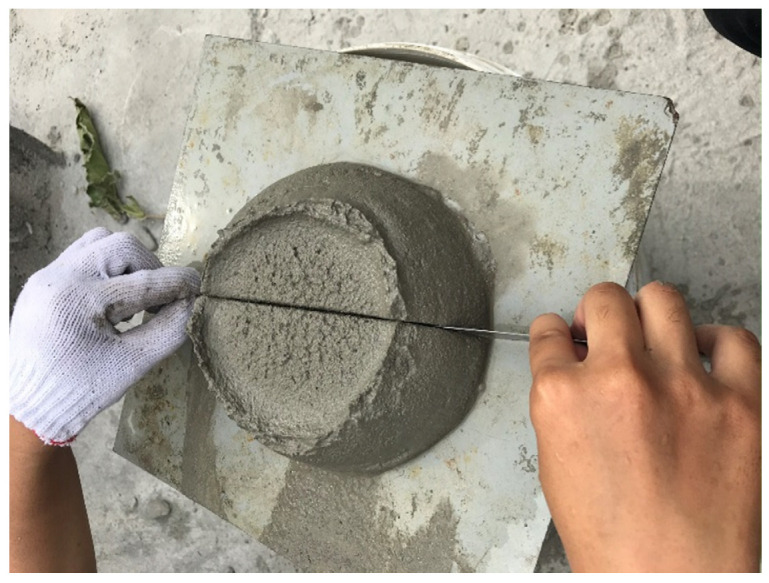
Measurement of the spreadability of foamed concrete.

This experiment was carried out to find out how adding PFF affected the foamed concrete flow and workability. Using an extended cylinder, spreadability was used to determine the workability (diameter of 76.2 mm × height of 152.4 mm). This apparatus was used following Brewer’s open-ended cylinder spread procedure [22,23]. Once the foamed concrete stopped flowing, the average spread diameter of the mixed foamed concrete was calculated. The measurement of foamed concrete’s spreadability is shown in Figure 3. 

### 3.2. Porosity Test

**Figure 4:**
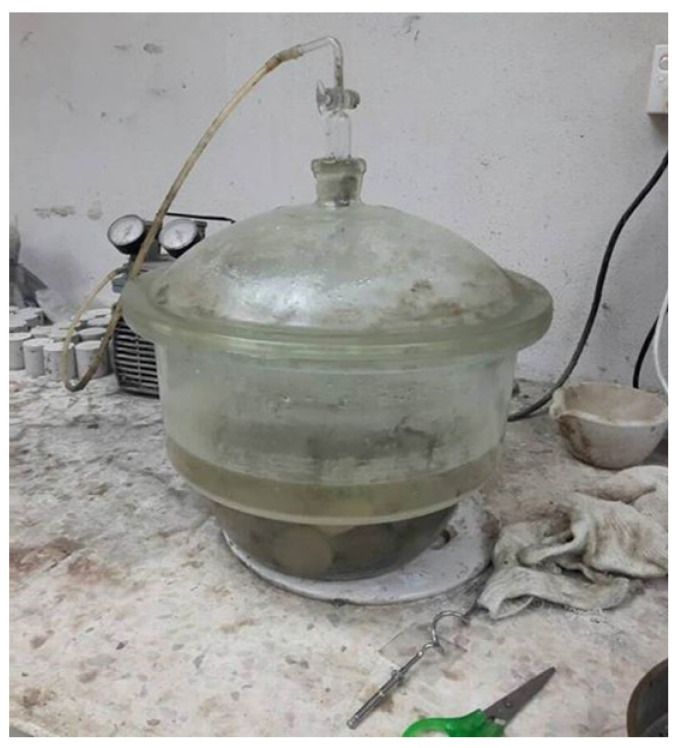
Vacuum saturation procedure.

The number of pores in foamed concrete is commonly stated as a percentage of volume. The porosity of foamed concrete affects many different aspects of its mechanical, thermal, and durability properties. Additionally, it allows several dangerous substances into the foamed concrete. Salt will corrode the reinforcing bar in reinforced foamed concrete, and rust will grow and cause the foamed concrete to crack. Determining the porosity of foamed concrete with a polyformaldehyde monofilament membrane is therefore critical. As shown in Figure 4, the vacuum-saturated method was used to conduct the porosity test [24]. The cylinder specimens used in casting had diameters of 45 mm and heights of 50 mm. On day 28, this test was executed by placing the foamed concrete samples inside a vacuum desiccator.

### 3.3. Water Absorption Test

The ability of foamed concrete to absorb water is directly related to its ability to repel water, something which is essential in many weakening mechanisms and prevents many harmful factors from entering the environment. The movement of oxygen, chlorine, and carbon dioxide, which start the corrosion of the reinforcing steel in foamed concrete, is made possible by the water absorption capacity of foamed concrete. The water absorption test was performed in this study in conformity with the BS 1881-122 specification [25]. Concrete cylinder specimens, 100 mm in height × 75 mm in diameter, were created.

### 3.4. Compression Test

**Figure 5:**
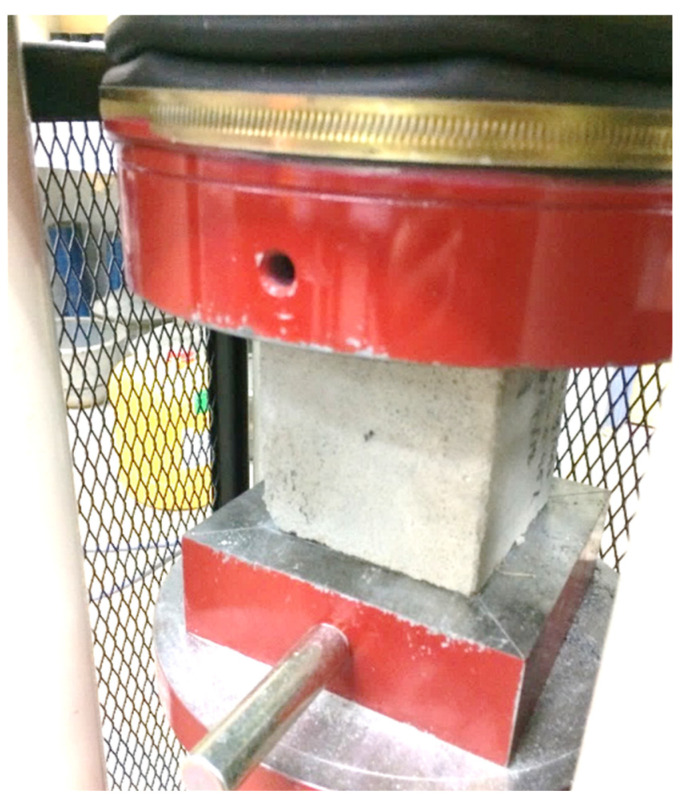
Setup for compression test.

The purpose of the axial compression test was to determine the potential strength of the foamed concrete mixtures from which the samples were taken. It evaluates the capacity of a foamed concrete specimen to withstand a load before failing. It permits the verification of whether the correct mix proportions of various foamed concretes and various ingredients were used to achieve the requisite strength. In this investigation, the axial compression test was conducted at a constant rate of 0.03 mm/s in accordance with the BS 12390-3 standard [26]. Cubic specimens with a dimension of 100 × 100 × 100 mm were prepared. The compression test was carried out on days 7, 28, and 56. The result was derived from the mean of three samples of foamed concrete. Figure 5 depicts the axial compression test configuration. 

### 3.5. Flexural Test

**Figure 6:**
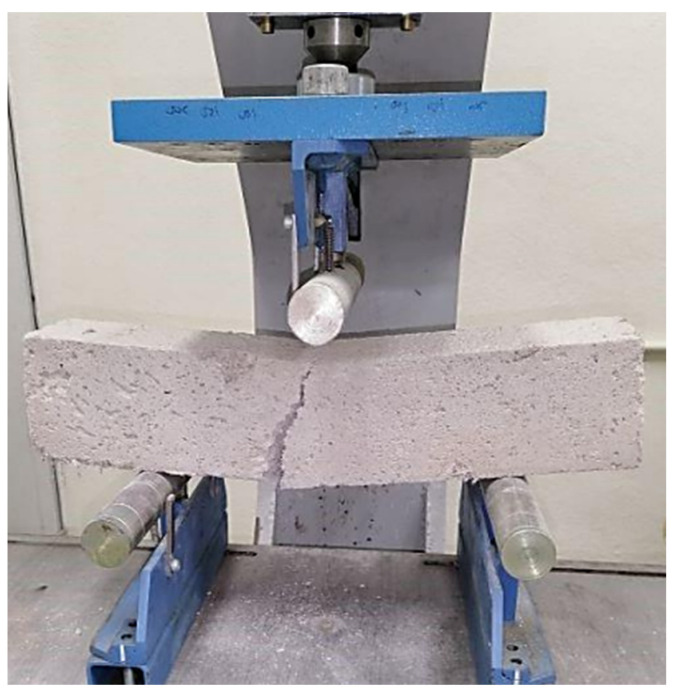
Apparatus for three-point bending test.

Flexural testing was performed to confirm the rigidity of foamed concrete and determine the force required to bend a prism. In a flexural test, ultimate stress represents the last stress level before failure. A three-point flexural test, as indicated in Figure 6, was undertaken in conformity with the BS EN 12390-5 standard [27] to assess the influence of PFF inclusion on the flexural strength of foamed concrete. Each flexural strength test is performed with 3 prisms that are each 100 mm × 100 mm × 500 mm in size and made from foamed concrete. Standard cure times of 7, 28, and 56 days were used for the flexural tests. 

### 3.6. Splitting Tensile Test

**Figure 7:**
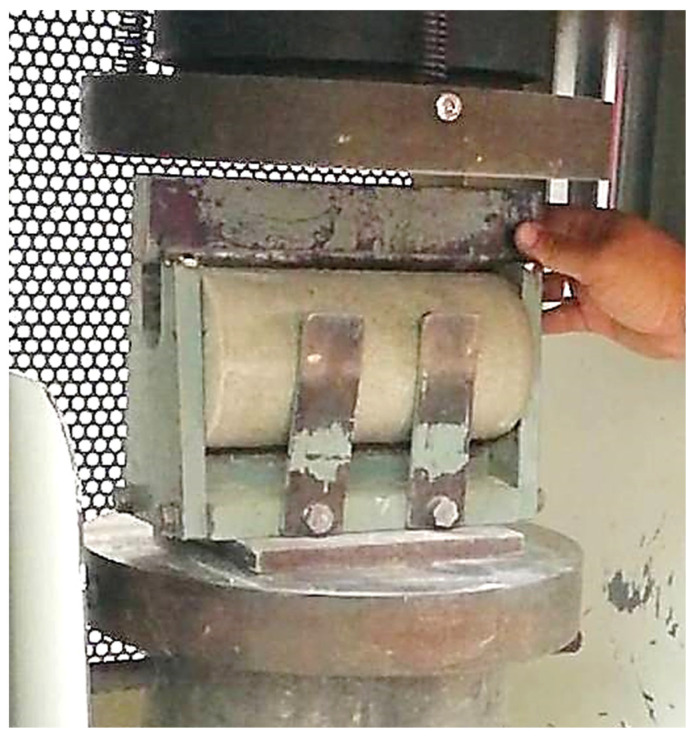
Splitting tensile test apparatus.

Foamed concrete’s splitting tensile strength is one of the essential and crucial qualities that significantly affects the depth and breadth of cracks in the material. Because of its brittleness and poor tensile strength, a foamed concrete specimen is not often anticipated to bear direct tension. In this research, the splitting tensile strength test was carried out in line with BS EN 12390-6 specifications [28]. The test was performed on a cylinder of foamed concrete, 100 mm in diameter by 200 mm in height, on days 7, 28, and 56. The splitting tensile strength test setup is displayed in Figure 7.

## 4. Discussion

### 4.1. Spreadability

**Figure 8:**
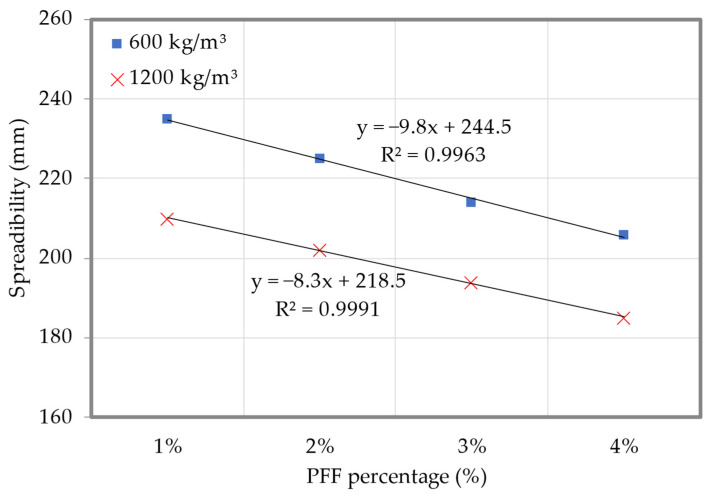
Spreadability of foamed concrete with varying percentages of PFF.

Figure 8 displays the results of the spreadability for both densities examined in this study (the dataset can be found in Appendix A, Table A2). Figure 8 shows that the self-flowing ability of all the foamed concrete mixes was excellent, with a spreadability greater than 187 mm. Spreadabilities of 255 mm and 230 mm were recorded for the 600 and 1200 kg/m^3^ densities of the control specimens, respectively. Spreadability was reduced by adding PFF to foamed concrete, and this effect was proportional to the amount of PFF added. Foamed concrete mixes with 4% PFF had the lowest spreadability when compared to the other foamed concrete mixes. Spreadabilities were measured to be 206 mm for 600 kg/m^3^ and 187 mm for 1200 kg/m^3^. Because PFF tends to absorb water, there is a consistent decrease in spreadability when it is are present; the external segment of PFF presents significant porosity, which benefits the bond to the matrices, and thus the spreadability of the foamed concrete decreases. In addition, the cementitious matrices aggregate on the PFF’s large specific surface area, increasing the foamed concrete’s viscosity and causing a decrease in spreadability at greater PFF weight fractions. As the air bubbles are forced out of the cementitious matrix, the slump diameter of the foamed concrete will decrease as a result of the free flow of the intermittent stage above the capacity of the stable spreading form. Mortar must additionally cover the smooth PFF membrane in addition to the fine filler (sand). The spreadability of foamed concrete was reduced as the percentage of PFF was raised from 1% to 4%, indicating that greater quantities filling mortar was required to cover the additional zone of PFF. Furthermore, PFF increases the inner abrasion between foamed concrete components, which in turn causes more cement matrix to diminish the inner resistance, hence reducing the workability of foamed concrete. A similar finding, that increasing the fiber’s weight fraction in concrete reduced its workability, was previously described by Mohseni et al. [29]. 

### 4.2. Density

**Figure 9:**
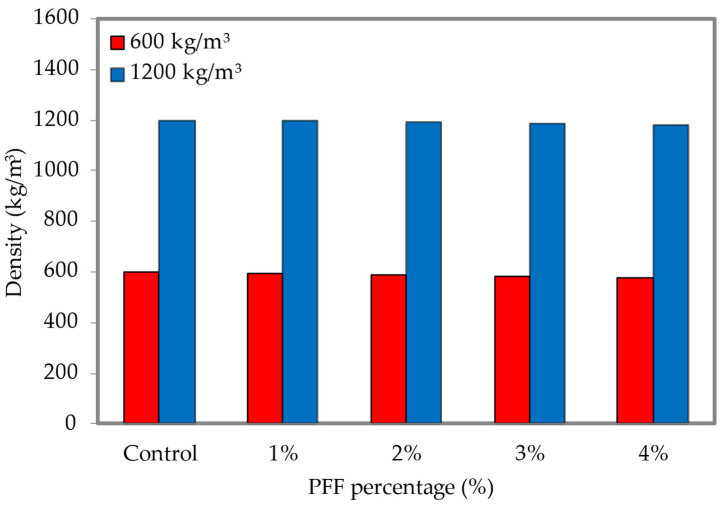
Density of foamed concrete with varying percentages of PFF.

Differences in the dry density of foamed concrete as a function of PFF weight % are shown in Figure 9 (the dataset can be found in Appendix A, Table A2). According to the findings, the dry density of foamed concrete decreases from that of the control foamed concrete across all three densities when the weight percentage of PFF increases from 1% to 4%. It was discovered that the foamed concrete dry density was highest when PFF was included at a rate of 0% (control specimen), and lowest when PFF was included at a rate of 4%. The decrease in dry density of LFC, combined with larger weight fractions of PF, was caused by difficulties in the compaction process. These resulted in absorbent LFC, resulting in a lower dry density of LFC as linked to the control specimen. Final dry densities were acceptable, falling within the range of 50 kg/m^3^ for all foamed concrete mixes that included PFF in different percentages. For instance, for mixes PF0%, PF1%, PF2%, PF3% and PF4%, the discrepancies between final dry densities and planned dry densities of 600 kg/m^3^ density were ±2 kg/m^3^, ±5 kg/m^3^, ±10 kg/m^3^, ±19 kg/m^3^ and ±24 kg/m^3^, respectively. The foamed concrete properties are entirely reliant on the dry density [30].

### 4.3. Porosity

**Figure 10:**
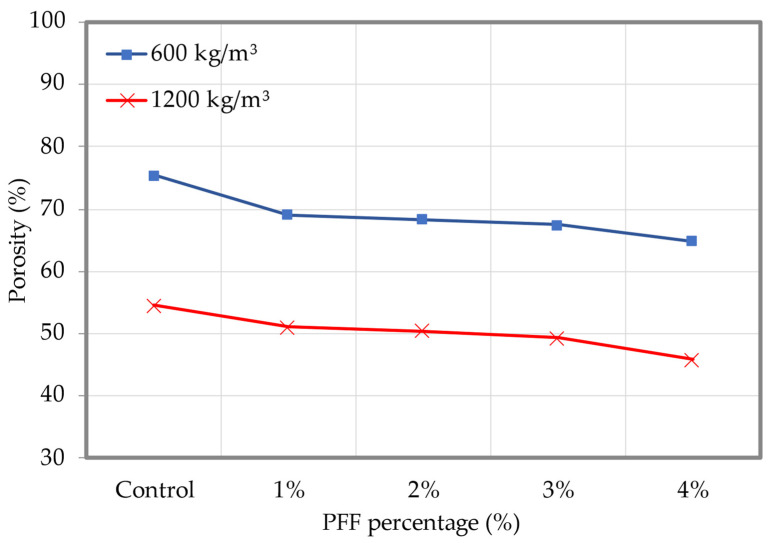
Porosity of foamed concrete with varying percentages of PFF.

**Figure 11:**
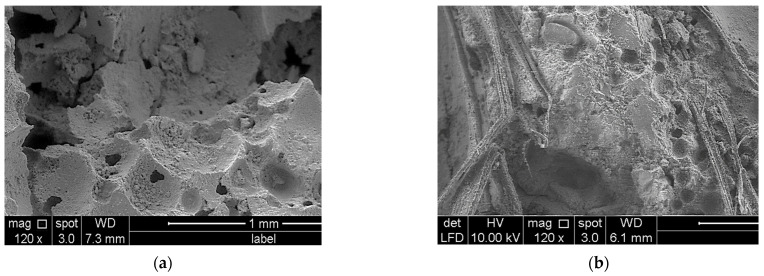
Morphology of (**a**) control specimen; (**b**) foamed concrete with 4% PFF.

The porosity of foamed concrete with increasing PFF percentages is represented graphically in Figure 10 (the dataset can be found in Appendix A, Table A2). Generally, the inclusion of PFF in foamed concrete results in a gradual increase in the material’s porosity, which reaches the highest value with a 4% addition of PFF. When contrasted with the control foamed concrete sample, the foamed concrete mix with 4% PFF obtained the optimal values of porosity, with around 13.9% and 15.9% decreases in porosity for 600 and 1200 kg/m^3^ densities, respectively. It is possible that this is because of the high packing capacity that PFF has in the cementitious matrix of foamed concrete. A porosity value of 64.9% was observed for the foamed concrete mix, with a density of 600 kg/m^3^ and a percentage of PFF of 4%, whereas a porosity value of 75.4% was recorded for the control sample. Microcracks formed on the surface of the foamed concrete while it was still in its fresh state condition. At the same time, the surface moisture was rapidly evaporating, which resulted in significant dry shrinkage. When PFF is added to foamed concrete mixes, the segregation can be reduced, and this also helps to reduce the amount of water that is lost through evaporation. In addition to this, the use of PFF has been shown to effectively stop the spread of cracks in foamed concrete that start on the surface and move inward [31]. The morphology of the control foamed concrete specimen, which has a density of 1200 kg/m^3^, is shown in Figure 11a. It was clear that there were a great number of huge pores that were attached to one another, which resulted in a high porosity value. With the presence of 4% PFF, the compactness of the foamed concrete is improved, and the number of pores that are both large and interconnected is reduced significantly. As the PF was incorporated into the cementitious matrix, as seen in Figure 11b, the internal structure became denser and a homogenous microstructure was achieved, all while decreasing the foamed concrete porosity value.

### 4.4. Water Absorption

**Figure 12:**
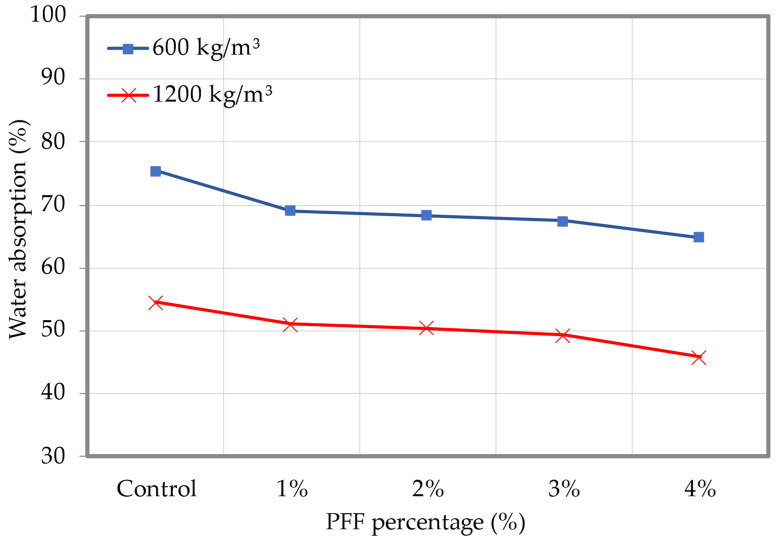
Water absorption of foamed concrete with varying percentages of PFF.

The findings of foamed concrete’s water absorption with various PFF percentages are shown in Figure 12 (the dataset can be found in Appendix A, Table A2). It was clear that as PFF percentages increased from 1% to 4%, the foamed concrete’s ability to absorb water increased as well. For both densities examined in this study, the inclusion of 4% of PFF resulted in the highest water absorption. It should be noted that less cracking occurs in foamed concrete when PFF is added to the mix, and the cracks that do form are smaller and finer than they are in foamed concrete without PFF. This suggests that the presence of PFF can significantly increase foamed concrete’s capacity to absorb water and significantly reduce the risk of microcrack merging [32]. Ramezanianpour et al. [33] explored the impact of polypropylene fiber percentages ranging from 0.5% to 4.0% on concrete water permeability. They discovered that adding polypropylene fiber decreased the extent of concrete water penetration. The effect of multi-size polypropylene fibers on the impermeability of concrete was analyzed by Guo et al. [34]. They discovered that, whereas coarse fibers had more obvious macropore inhibition effects on concrete, small fibers had noticeable inhibition on micropores. Additionally, the impenetrability of foamed concrete made with coarse and fine polypropylene fiber is higher than that of foamed concrete made with single-diameter polypropylene fiber. Behfarnia and Behravan [35] evaluated the concrete’s ability to absorb water between 0.4% and 0.8% steel fiber and polypropylene fiber. They discovered that the presence of polypropylene fiber diminished the concrete’s capacity to absorb water by up to 45%, indicating that concrete’s impermeability was greatly increased. 

### 4.5. Water Absorption—Porosity Relationship

**Figure 13:**
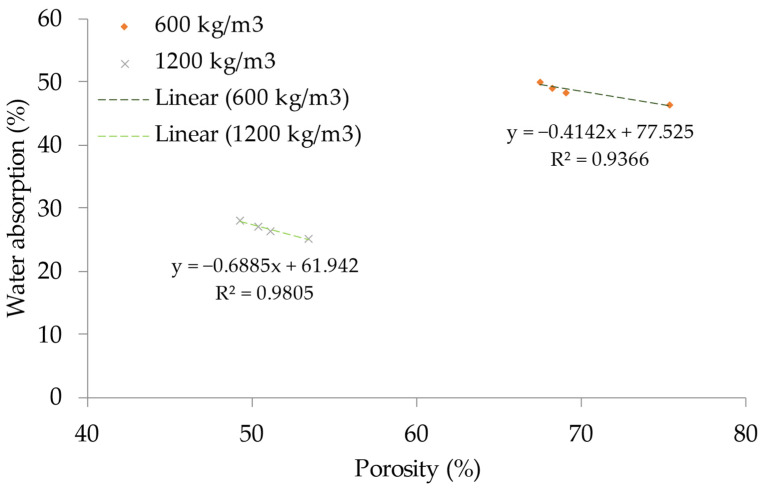
Relationship between water absorption and porosity.

The correlation between foamed concrete water absorption capacity and its level of porosity is seen in Figure 13. When the percentage of PFF changes, a linear relationship develops between the foamed concrete water absorption and porosity. This relationship states that the foamed concrete porosity will expand in tandem with the foamed concrete water absorption as the water absorption of the foamed concrete increases. The outward area of the foamed concrete is where the dispersal of free water first begins, eventually making its way into the foamed concrete matrix’s deepest segment. In addition, the amount of water that is absorbed internally by foamed concrete may have a marginal impact on the porosity of the material. The fact that there is a definite linear association between the water-absorbing capacity of foamed concrete and its porosity can be seen from the fact that its R-squared values are 0.9805 and 0.9366 for 1200 and 600 kg/m^3^ densities, correspondingly.

### 4.6. Compressive Strength

**Figure 14:**
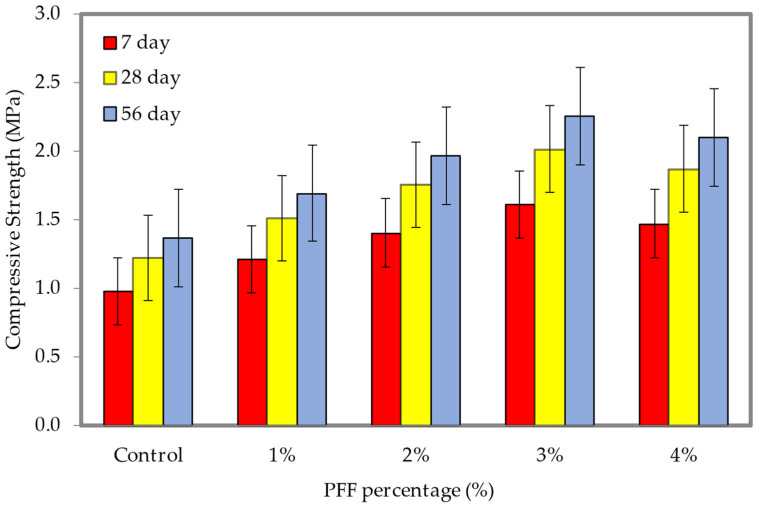
Compressive strength of foamed concrete of 600 kg/m^3^ density with varying PFF percentages.

**Figure 15:**
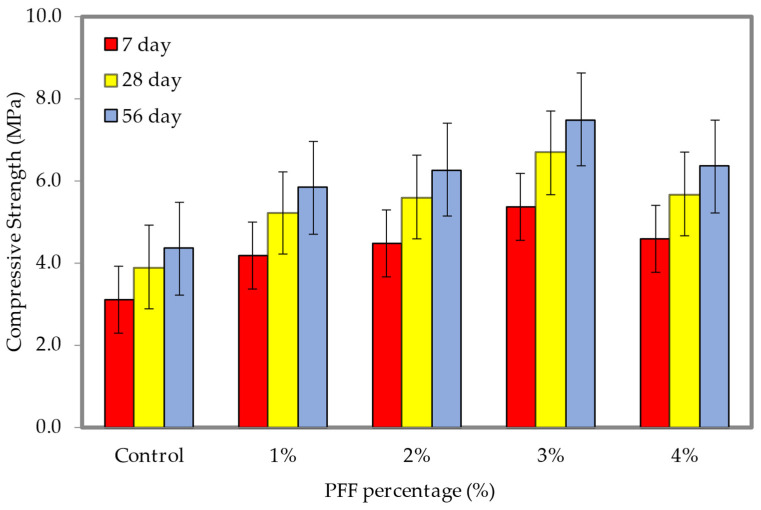
Compressive strength of foamed concrete of 1200 kg/m^3^ density with varying PFF percentages.

The compressive strength results of 600 and 1200 kg/m^3^ densities, corresponding with the insertion of various percentages of PFF, are shown in Figure 14 and Figure 15 (the dataset can be found in Appendix A, Table A1). Generally, adding PFF to foamed concrete limited in the growth of the material’s compressive strength. For both densities, the optimal percentage of PFF was 3%. PFF was added to foamed concrete, which resulted in a decrease in the amount of entrapped air voids, capillary pores, and entrained air voids. All three of these factors contribute to a rise in the foamed concrete compressive strength. The compressive strengths of the foamed concrete on days 7, 28, and 56 with the inclusion of PFF were greater than the control specimen strength, regardless of the density of the foamed concrete. In comparison to the control specimen, which only achieved compressive strengths of 1.37 MPa (600 kg/m^3^) and 4.34 MPa (1200 kg/m^3^), the optimal compressive strengths achieved on day 56 were 2.26 MPa and 7.83 MPa, with the inclusion of a 3% and 4% weight fraction of PFF for the 600 and 1200 kg/m^3^ densities, respectively. When an optimal percentage of PFF is evenly scattered in foamed concrete cement paste, the hydrated products of cement amass around the PFF. This is due to their superior surface energy, as their surface behaves as a nucleation site. As the foamed concrete contracts, the PFF membrane absorbs tensile energy through the boundary between the PFF and the foamed concrete cementitious matrix. It then transfers this energy to the neighboring matrix, thereby reducing the amount of concentrated tensile stress and increasing the foamed concrete’s resistance to cracking [36]. Poor PFF dispersal in the foamed concrete cementitious matrix results in a balling effect when the percentage of PFF in the foamed concrete exceeds 3%. This creates a deteriorated impact by scattering the tensile stress from the vicinity of the foamed concrete to another place on the PFF surface. This explanation lends credence to the idea that a decline in compressive strength occurred when the weight fractions of PFF increased to a level greater than 3%. 

### 4.7. Flexural Strength

**Figure 16:**
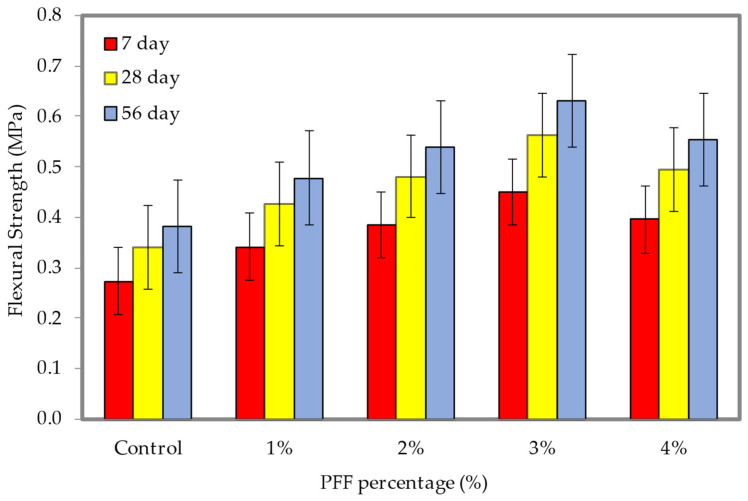
Flexural strength of foamed concrete of 600 kg/m^3^ density with varying PFF percentages.

**Figure 17:**
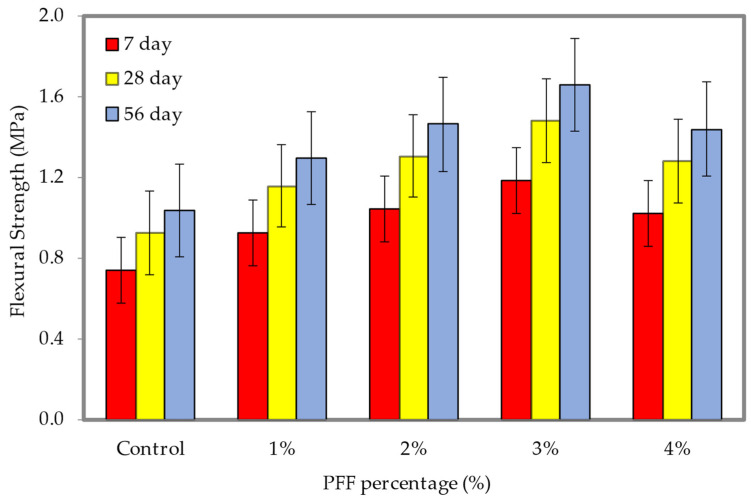
Flexural strength of foamed concrete of 1200 kg/m^3^ density with varying PFF percentages.

The results of the flexural strength tests at densities of 600 and 1200 kg/m^3^ are shown in Figure 16 and Figure 17, respectively (the dataset can be found in Appendix A, Table A1). These densities correspond with different weight fractions of PFF. It is clear from looking at Figure 16 and Figure 17 that the inclusion of PFF in foamed concrete led to a rise in flexural strength. For both densities considered in this study, the best weight fraction of PFF was 3%. The flexural strengths of the foamed concrete with the presence of PFF on days 7, 28, and 56 were greater than the flexural strength of the control specimen, regardless of the density of the foamed concrete. In contrast to the control specimen, which only achieved flexural strengths of 0.38 MPa (600 kg/m^3^) and 1.04 MPa (1200 kg/m^3^), the ideal flexural strengths that were attained at day 56 were 0.63 MPa and 1.66 MPa, with the addition of a 3% of PFF for the 600 kg/m^3^ and 1200 kg/m^3^. PFF is a hydrophilic substance, and as a result, it possesses great adhesion when combined with cement paste. After the PFF percentage was increased to 4%, the flexural strength of foamed concrete drastically dropped for both densities that were taken into consideration for this investigation. Because it is difficult to disperse the PFF evenly and because it might cause agglomeration, the flexural strength will be lowered if the percentage of PFF is too high. This is because PFF can cause agglomeration. During the process of the crack spreading out, the PFF will gradually become separated from the matrix until the bond strength is completely exceeded. This will continue until the fracture has completely spread out. Although the matrix is damaged, it is still capable of maintaining its fundamental form. The presence of PFF in foamed concrete plays a significant role in both the strengthening of the foamed concrete cementitious matrix, and the modification of the material’s physical characteristics from a brittle state to a ductile one. Both of these effects are the result of the material’s transition from a brittle to a ductile state. Because an appropriate percentage of PFF can bond with the hydration products and unhydrated constituents in the foamed concrete matrix to build a three-dimensional grid structure, incorporating an appropriate weight fraction of PFF can efficiently increase the flexural strength of foamed concrete. This is because the three-dimensional network structure can create a subsidiary effect and boost the foamed concrete flexural strength [37]. 

### 4.8. Splitting Tensile Strength

**Figure 18:**
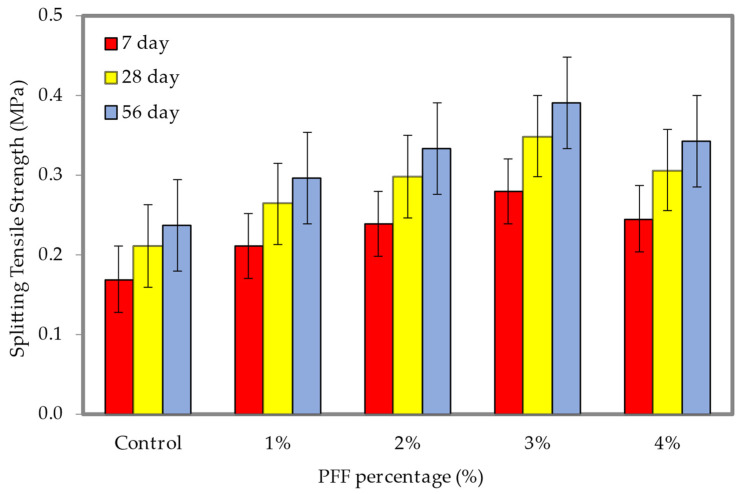
Splitting tensile strength of foamed concrete of 600 kg/m^3^ density with varying PFF percentages.

**Figure 19:**
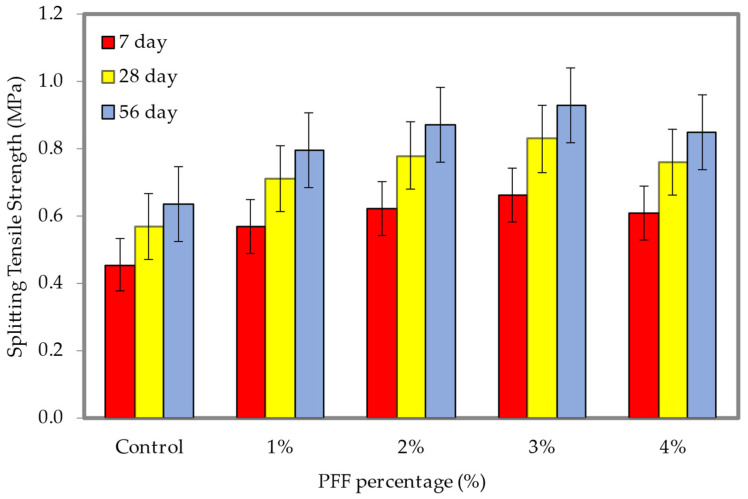
Splitting tensile strength of foamed concrete of 1200 kg/m^3^ density with varying PFF percentages.

Figure 18 and Figure 19 illustrate the influence that different percentages of PFF have on the splitting tensile strength with densities of 600 and 1200 kg/m^3^ (the dataset can be found in Appendix A, Table A1). It is clear from the whole trend that with the rise in PF percentages, the splitting tensile strength of both foamed concrete densities at different ages all exhibit an upward trend. This is the case because the PFF percentages are increasing as the trend continues. In most cases, the splitting tensile strength progressively increases with the growth in the percentages of PFF up to 3%. In comparison to the control specimen, which achieved compressive strengths of 0.24 MPa (600 kg/m^3^) and 0.64 MPa (1200 kg/m^3^), the optimal splitting tensile strengths attained at day 56 were 0.39 MPa and 0.93 MPa, with the inclusion of a 3% PFF for the 600 and 1200 kg/m^3^ densities. Because of the enhanced foamed concrete robustness, helped along by the presence of PFF, the enhancement of splitting tensile strengths was accomplished for both materials that were taken into consideration throughout this investigation. PFF will gradually form a fiber grid skeleton inside the foamed concrete as it goes through the process of hardening. This can improve the brittle state of the foamed concrete matrix by governing the growth and propagation of cracks when it is subjected to external tensile stress, preventing the cracks from leading to an explosive failure. It became apparent that the PFF could be uniformly disseminated when the PFF percentage was 3%, which resulted in a growth in the bonding strength between the cement matrix and the PFF. The PFF is dispersed throughout the foamed concrete matrix in a manner that is nearly homogeneous and does not show any significant signs of buildup. In the presence of a tensile load, foamed concrete demonstrates elastic linear tension prior to the onset of plastic deformation, which occurs after the appearance of the first crack in the material. The addition of PFF membrane to foamed concrete, on the other hand, causes the membrane to take on the role of fastening when the foamed concrete cracks. This ensures that the matrix elastic modulus does not immediately drop to zero when the direct boundary strain is reached [38,39,40]. When cracks appear in the PFF membrane, the membrane will bear all the tension and then gradually transmit it to the matrix [8,41,42,43,44,45]. 

### 4.9. Compressive—Flexural Strengths Relationship

**Figure 20:**
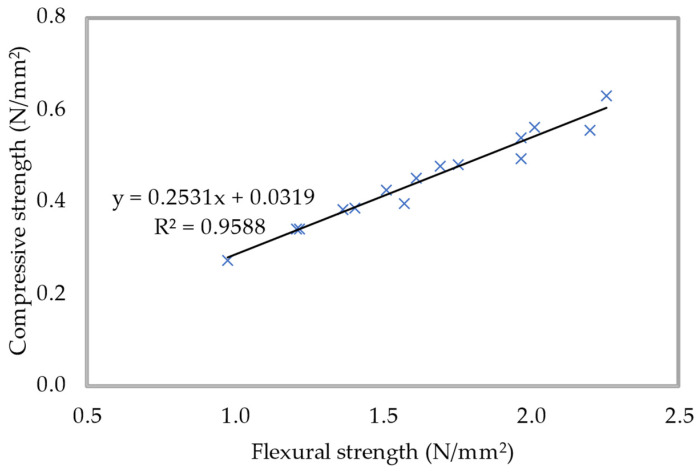
Flexural-compressive strengths relationship for 600 kg/m^3^ density.

The density of 600 kg/m^3^ of foamed concrete was used in the execution of a correlation between the compressive and flexural strengths of the material. For the purpose of this investigation, all curing periods were taken into account. The connection between the flexural and compressive strengths of foamed concrete with various percentages of PFF is demonstrated in Figure 20. It seems that they found out that there is a direct expanding, relationship that can be differentiated in the compressive strength and flexural strength of foamed concrete. From Figure 20, it can be seen that an R-squared value of 0.9588 was obtained, which indicates a highly linear relationship between the two strength parameters. This implies that variations in the predictors are interrelated to deviations in the response variable and that the achieved prediction models explain a significant portion of the response’s inherent variability. Because of this relationship, it is clear that the flexural strength of foamed concrete increases as the compressive strength of the concrete increases. This regression model makes it possible to approximate the flexural strength of foamed concrete, based on its axial compressive strength, for the range of values that was investigated in this exploration.

### 4.10. Compressive—Splitting Tensile Strengths Relationship

**Figure 21:**
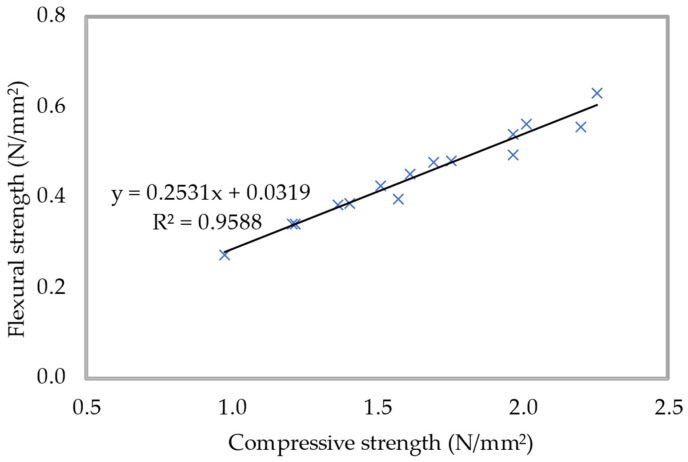
Compressive-tensile strengths relationship for 600 kg/m^3^ density.

The relationship between the splitting tensile and compressive strengths of 600 kg/m^3^ density foamed concrete with different PFF percentages is shown in Figure 21. The compressive strength was mapped against the foamed concrete’s splitting tensile strength. According to Figure 21, data dissemination supports the existence of a strong correlation between the splitting tensile and compressive strengths of foamed concrete. In a similar trend, the splitting tensile strengths rose with increasing compressive strength for all curing periods. With an R-squared value of 0.9646, a strong linear connection is evident. The splitting tensile strength was approximately 15% of its strength under compression conditions for the total specimens evaluated in this investigation.

## 5. Conclusions

This laboratory investigation aims to evaluate the possible use of polypropylene fibrillated fiber (PFF) to enhance the foamed concrete engineering properties. With the inclusion of four different PFF percentages (1%, 2%, 3%, and 4%), ten mixes of 600 and 1200 kg/m^3^ densities were made. Compressive, splitting tensile and flexural strengths were assessed. Additionally, the workability, porosity, water absorption, and density of products were evaluated as well. Additionally, the correlations between the considered properties were analyzed. When PFF is added to foamed concrete, spatial networks are formed and cement paste is incinerated to cover the PFF, resulting in reduced spreadability versus foamed concrete without fibers. However, all foamed concrete mixes exhibited spreadabilities greater than 187 mm, indicating a significant capacity for self-flow. In contrast to the control specimen, the dry density of the foamed concrete decreases as the PFF weight fractions rise from 1% to 4%. The control specimen had the highest dry density of foamed concrete, whereas the 4% PFF inclusion product had the lowest density. Due to the intricacy of the compaction process, which produces porous foamed concrete, the dry density decreased at larger weight fractions of PFF. With the presence of PFF, the foamed concrete porosity grows gradually up to 4%. Foamed concrete mixes containing 4% PFF achieved the optimal porosity with a reduction of around 13.9% and 15.9% for 600 and 1200 kg/m^3^ densities correspondingly. This is most likely because foamed concrete’s cement matrix has strong PFF packing capabilities. As the PFF percentages rose from 1% to 4%, foamed concrete water absorption was increased. With the addition of 4% PFF, the highest water absorption capacity was achieved. When PFF is added to foamed concrete, the fissures are less noticeable and finer than they are in foamed concrete that does not contain PFF. The foamed concrete flexural, compressive, and splitting tensile strengths were increased by the addition of PFF. The ideal percentage of PFF for both densities was 3%. The foamed concrete’s compressive, flexural, and breaking tensile strengths considerably decreased above the 3% PFF. It is difficult to spread the PFF uniformly, and agglomeration is caused if the PFF weight fractions are too large.

## Figures and Tables

**Table 1:** Physical and mechanical attributes of PFF.

Properties	Value
Elastic modulus (GPa)	7.25
Tensile strength (MPa)	915
Elongation at failure (%)	19.3
Thermal conductivity (W/mK)	0.255
Specific heat capacity (J/kgK)	1335
Melting temperature (°C)	160
Specific weight (g/cm^3^)	0.89
Thickness (mm)	0.25
Length (mm)	19

**Table 2:** Mixture proportions of foamed concrete.

Density (kg/m^3^)	PFF (%)	Cement (kg/m^3^)	Sand (kg/m^3^)	Water (kg/m^3^)	Foam (kg/m^3^)	PFF (kg/m^3^)
600	0	230.2	345.4	103.6	43.2	0.0
600	1	230.2	345.4	103.6	43.2	7.2
600	2	230.2	345.4	103.6	43.2	14.4
600	3	230.2	345.4	103.6	43.2	21.7
600	4	230.2	345.4	103.6	43.2	28.9
1200	0	446.9	670.4	201.1	24.4	0.0
1200	1	446.9	670.4	201.1	24.4	13.4
1200	2	446.9	670.4	201.1	24.4	26.9
1200	3	446.9	670.4	201.1	24.4	40.3
1200	4	446.9	670.4	201.1	24.4	53.7

## Appendix A

**Table A1:** Compressive, flexural, and splitting tensile strengths experimental dataset.

Specimen		600 kg/m^3^			1200 kg/m^3^		
		7 Day	28 Day	56 Day	7 Day	28 Day	56 Day
Compressive Strength (MPa)	Control	0.98	1.22	1.37	3.11	3.88	4.35
	1%	1.21	1.51	1.69	4.16	5.20	5.83
	2%	1.40	1.76	1.97	4.47	5.59	6.26
	3%	1.61	2.02	2.26	5.34	6.68	7.48
	4%	1.47	1.87	2.10	4.57	5.67	6.35
Flexural Strength (MPa)	Control	0.27	0.34	0.38	0.74	0.93	1.04
	1%	0.34	0.43	0.48	0.93	1.16	1.30
	2%	0.38	0.48	0.54	1.04	1.31	1.46
	3%	0.45	0.56	0.63	1.19	1.48	1.66
	4%	0.40	0.49	0.55	1.02	1.28	1.44
Splitting Tensile Strength (MPa)	Control	0.17	0.21	0.24	0.46	0.57	0.64
	1%	0.21	0.26	0.30	0.57	0.71	0.80
	2%	0.24	0.30	0.33	0.62	0.78	0.87
	3%	0.28	0.35	0.39	0.66	0.83	0.93
	4%	0.25	0.31	0.34	0.61	0.76	0.85

**Table A2:** Slump, density, porosity, and water absorption experimental dataset.

Specimen		600 kg/m^3^	1200 kg/m^3^
Workability (mm)	Control	255	230
	1%	235	210
	2%	225	202
	3%	214	194
	4%	206	185
Density (kg/m^3^)	Control	600	1200
	1%	594	1195
	2%	589	1190
	3%	580	1185
	4%	574	1181
Porosity (%)	Control	75.4	54.5
	1%	69.1	51.1
	2%	68.3	50.4
	3%	67.5	49.3
	4%	64.9	45.8
Water Absorption (%)	Control	46.4	25.9
	1%	48.4	26.5
	2%	49.2	27.3
	3%	50.0	28.1
	4%	50.7	29.2
